# Yellow Nail Syndrome: A Differential Diagnosis That Must Be in Mind

**DOI:** 10.7759/cureus.95953

**Published:** 2025-11-02

**Authors:** Inês Genrinho, Mariana Diz-Lopes, António Morais, Iva Brito

**Affiliations:** 1 Rheumatology, Unidade Local de Saúde Viseu Dão Lafões, Viseu, PRT; 2 Rheumatology, Unidade Local de Saúde de São João, Porto, PRT; 3 Pulmonology, Unidade Local de Saúde de São João, Porto, PRT; 4 Faculty of Medicine, University of Porto, Porto, PRT; 5 Pediatrics and Young Adult Rheumatology, Unidade Local de Saúde de São João, Porto, PRT

**Keywords:** lymphedema, onycholysis, recurrent pneumonia, systemic sclerosis, yellow nails

## Abstract

Yellow nail syndrome (YNS) is a rare disorder that consists of a clinical triad of thickened yellow nails, lymphedema, and recurring respiratory symptoms. These findings can occur simultaneously or additively, and the characteristic nail changes are an obligatory feature for the diagnosis. The diagnosis of YNS is one of exclusion, and although it is more often an isolated condition, it can occur secondary to immune or neoplastic disorders. Involvement of the respiratory tract occurs in most cases and has a diverse clinical presentation, including pleural effusion and recurrent pneumonia. Due to the rarity of this syndrome and the limited availability of effective treatments, YNS remains associated with substantial morbidity. We present a case of a 58-year-old woman who had a history of recurrent pneumonia, chronic sinusitis, arthralgias, and yellow dystrophic nails that was diagnosed with YNS, and in which daily supplementation with vitamin E resulted in a remarkable clinical improvement.

## Introduction

Yellow nail syndrome (YNS) is a rare disorder, with an estimated prevalence <1/1,000,000 [1]. It can affect both sexes and usually occurs after 50 years old, with pediatric forms being rarely reported [2-4].

YNS is charac­terized by a clinical triad consisting of thickened yellow nails, lymphedema, and recurring respiratory manifestations [5]. These three clinical characteristics do not always manifest simultaneously; they may appear individually and sequentially with intervals of several years. The diagnosis requires the presence of characteristic nail changes, along with at least one of the other two components of the triad [6].

The pathogenesis of YNS remains unclear. Leading theories suggest lymphatic dysfunction [7], microvasculopathy with protein leakage [8], and, more recently, certain environmental exposures - particularly titanium [9].

YNS is most often an isolated condition; however, previous reports have described associations with autoimmune diseases, disorders affecting lymphatic function, and cancers, supporting its classification as a paraneoplastic syndrome [1].

Due to the rarity of this syndrome, the diagnosis is often delayed. Early identification of YNS is important to monitor and control respiratory problems and associated disorders [8]. The disease has a high morbidity due to the lack of definitive therapies, and its management is mainly supportive and palliative [10].

## Case presentation

A 58-year-old woman with a history of paroxysmal atrial fibrillation and prior sinus surgery with septoplasty at the age of 18 was referred to the Rheumatology Department due to a 14-month history of recurrent pneumonia, arthralgias, and yellow dystrophic nails.

The patient experienced menopause at the age of 55, was a non-smoker, had no history of occupational exposure to harmful agents, and had no reported allergies. Her current medications included a budesonide/formoterol/glycopyrronium bromide inhaler, as well as a combination of 500 mg of naproxen and 20 mg of esomeprazole for arthralgia episodes. She had a history of treatment with various antifungal agents, with no benefit in the nail involvement.

Respiratory symptoms and nail discoloration had begun simultaneously 14 months ago, accompanied by chronic sinusitis, which required multiple courses of antibiotics and corticosteroid therapy. The diffuse arthralgias occurred only during episodes of increased body temperature associated with recurrent infectious intercurrences, improving with anti-inflammatory treatment and after resolution of the underlying condition.

Upon inquiry, the patient denied specific symptoms suggestive of connective tissue diseases, including Raynaud’s phenomenon, dysphagia, or skin thickening. She reported lower limb edema only recently, prior to the consultation evaluation.

**Figure 1:**
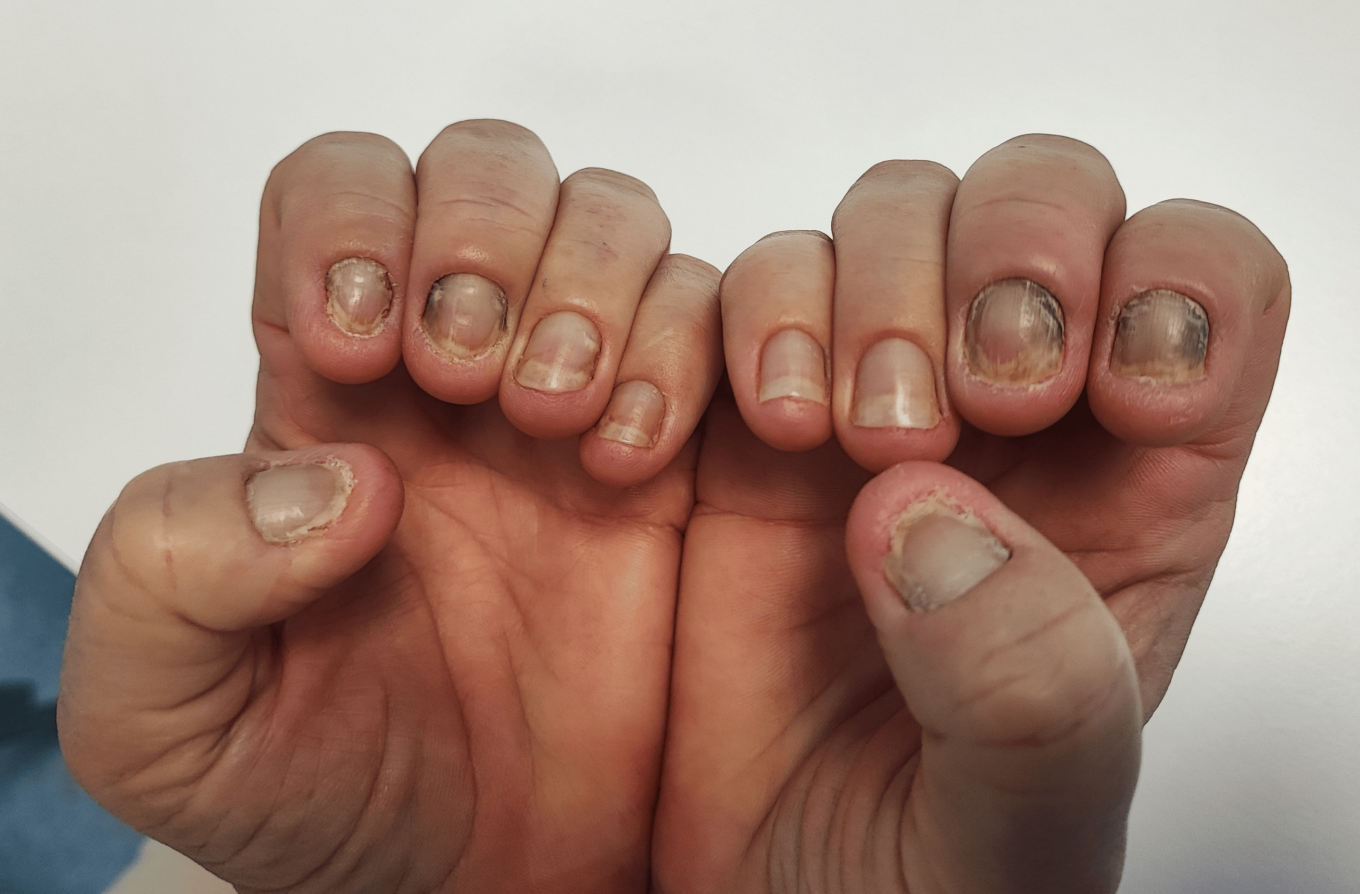
Yellow dystrophic nails involving all the fingers. Typical signs: absence of the cuticles, yellow discoloration, and increased transverse curvature.

**Figure 2:**
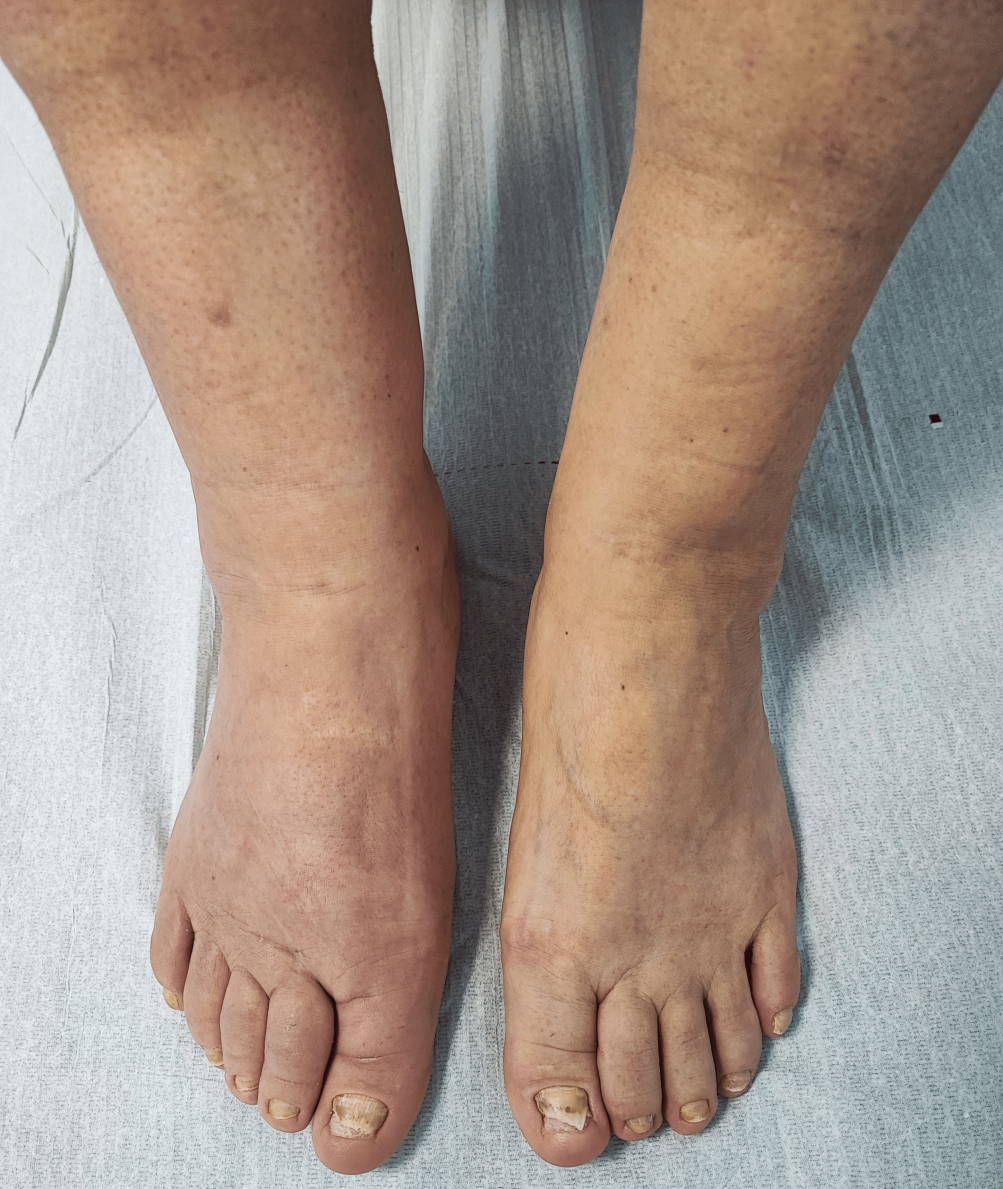
Lymphedema and dystrophic yellowish toenails.

The musculoskeletal examination was unremarkable, with the exception of thick and yellowish discoloration, with a lack of the cuticle of the digital and toenails (Figures 1-2). The patient was also found to have bilateral pitting lower extremity edema (Figure 2).

**Figure 3:**
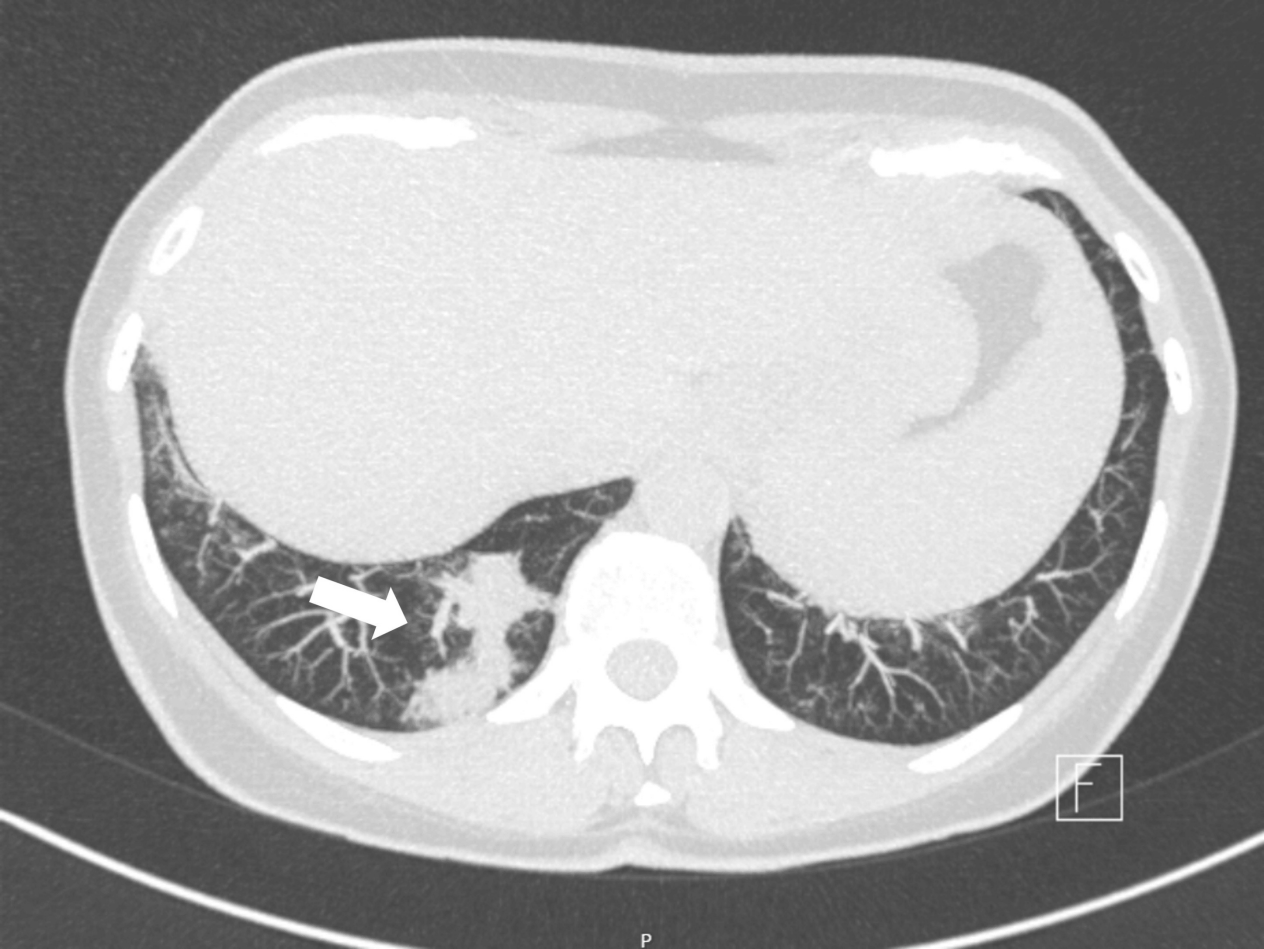
Axial HRCT showing small consolidative foci in the right lower lobe (white arrow), consistent with organizing pneumonia. HRCT: High-resolution computed tomography

The chest radiograph showed no significant abnormalities. High-resolution computed tomography (HRCT) of the lungs revealed small consolidative foci in multiple regions (Figure 3), consistent with pneumonia. Histological analysis of the CT-guided transthoracic biopsy revealed a nonspecific reactive chronic inflammatory infiltrate, a global preservation of normal architecture, and no signs of fibrosis. Pulmonary function tests showed an obstructive pattern, with forced vital capacity (FVC) of 85.3%, forced expiratory volume (FEV1) of 78.6%, and the FEV1/FVC ratio of 69.6%.

Among the other imaging studies, the presence of a multinodular thyroid with benign histology was noted, and the abdominal-pelvic CT and transthoracic echocardiogram showed no significant abnormalities.

The analytical workup revealed no abnormalities in the complete blood count, renal and liver function, or calcium-phosphate metabolism. There was a mild elevation in inflammatory markers, with an erythrocyte sedimentation rate (ESR) of 30 mm/h and C-reactive protein (CRP) of 8.4 mg/L. Autoimmunity testing was negative for antinuclear antibodies, anti-double-stranded DNA, extractable nuclear antigen, antineutrophil cytoplasmic antibodies, antiphospholipid antibodies, rheumatoid factor, anti-cyclic citrullinated peptide, and HLA-B27, with normal complement levels and immunoglobulins. Only a positive result for anti-Scl70 antibody was identified in the systemic sclerosis (SSc) panel, accompanied by an increase in thyroid antibodies, though thyroid function remained normal. Serological tests were negative for toxoplasmosis, rubella, *Brucella, Borrelia, Cytomegalovirus,* Epstein-Barr virus, Herpes simplex, human immunodeficiency virus, hepatitis B and C virus, and infectious screening for tuberculosis and syphilis (Table 1).

**Table 1:** Summary of immunologic and infectious laboratory investigations from the present clinical case.

Test	Result	Reference range
White blood cell count	8.64 x 10^9^	4.00 - 11.00 x 10^9^/L
% neutrophils	67.1	53.8 - 69.8%
Hemoglobin	12.9	12.0 - 16.0 g/dL
Hematocrit	37.8	36 - 46%
Platelet	288 x 10^9^	150 - 400 x 10^9^/L
Erythrocyte sedimentation rate	30 mm/h	0 - 20 mm/h
Creatinine	0.65	0.51 - 0.95 mg/dL
Aspartate aminotransferase	18	10 - 31 U/L
Alanine aminotransferase	12	10 - 31 U/L
Albumin	40.6	38 - 51 g/L
Lactate dehydrogenase	144	135 - 225 U/L
Calcium	4.8	4.2 - 5.1 mEq/L
Inorganic phosphorus	3.9	2.7 - 4.5 mg/dL
Magnesium	1.49	1.55 - 2.05 mEq/L
C-reactive protein	8.4	<3.0 mg/L
C3 level	178	82 - 170 mg/dL
C4 level	32.9	12 - 36 mg/dL
Immunoglobulin A	256	78 - 312 mg/dL
Immunoglobulin M	161	55 - 300 mg/dL
Immunoglobulin G	1300	650 - 1500 mg/dL
Thyroid-stimulating hormone	1.110	0.55 - 4.78 UI/mL
Free thyroxine	1.11	0.89 - 1.76 ng/dL
Antinuclear antibodies	Negative	< 1/160
Anti-double stranded DNA	24.1	< 25 UI/mL
Extractable nuclear antigen	Negative	
Systemic sclerosis panel	Anti-Scl70+++	
Antineutrophil cytoplasmic antibodies (PR3 and MPO)	2	< 20 U/mL
Antiphospholipid antibodies (lupus anticoagulant, anticardiolipin IgM and IgG, anti-β2 glycoprotein I IgM and IgG)	Negatives	
Rheumatoid factor	9.7	< 30 UI/mL
Anti-cyclic citrullinated peptide	7.2	< 20 U/mL
HLA-B27	Negative	
Anti-thyroglobulin	55.7	< 4.1 UI/mL
Anti-thyroid peroxidase	60.3	< 5.6 UI/mL
Serological tests (Toxoplasmosis, Rubella, Brucella, Borrelia, Cytomegalovirus, Epstein-Barr virus, Herpes simplex, human immunodeficiency virus, hepatitis B and C virus)	Negatives	
Interferon-gamma release assay	Negative	
Venereal disease research laboratory test	Negative	

Based on the clinical presentation with characteristic nail involvement, recurrent respiratory infections in the form of organizing pneumonia, chronic sinusitis, and, more recently, lymphedema, and, after exclusion of autoimmune diseases, a diagnosis of YNS was assumed. At her six-month follow-up evaluation, she reported significant improvement in nail dystrophy with daily vitamin E supplementation (1.125 IU/day). Additionally, a prophylactic dose of azithromycin, three times per week, was initiated by her pulmonologist. Despite the positivity for anti-Scl70, the patient currently does not fulfill the clinical criteria for a definitive diagnosis of SSc. Pulmonary involvement related to SSc could not be entirely ruled out, and a regular follow-up assessment by a multidisciplinary team is recommended to monitor disease progression and to consider potential therapeutic adjustments.

Written informed consent was obtained from the patient for publication of this case and any accompanying images.

## Discussion

First described in 1894 and later named by Samman and White in 1964 [7], YNS was characterized by Emerson as a triad consisting of nail abnormalities, lymphedema, and pleural effusions [11]. Over time, the diagnostic criteria were broadened to encompass additional chronic respiratory manifestations, such as sinusitis, bronchitis, recurrent pneumonias, pleuritis, and bronchiectasis [5].

YNS is a clinical diagnosis that is established by exclusion. The diagnosis is established based on the presence of characteristic nail changes in combination with at least one of the other two triad components-lymphedema or respiratory symptoms [6].

The classic three symptoms do not always manifest simultaneously, and individual features may emerge years apart. However, similar to the present report, nail changes typically develop after pulmonary abnormalities [4].

YNS is rarely congenital or seen in pediatric patients, and it predominantly affects middle-aged adults, with both males and females being equally affected [2-4].

The pathophysiology of YNS remains unknown, although several hypotheses have been proposed. Impaired lymphatic drainage is frequently cited as a central factor contributing to lymphedema, pleural effusion, and nail changes. However, Maldonado et al. [8] have suggested that protein leakage secondary to microvasculopathy may play a more prominent role than lymphatic dysfunction. Supporting this hypothesis, some reports have described dilated and tortuous capillary loops on nailfold capillaroscopy [12].

More recently, a potential association between titanium exposure - particularly titanium dioxide - and YNS has been proposed. Proposed sources of titanium include dental and orthopedic implants, surgical staples, pharmaceutical excipients, certain foods (chewing gum, particularly in pediatric cases), and cosmetic products such as sunscreens, moisturizers, shampoos, and toothpastes [13,14].

Among the several clinical manifestations, yellow nails are the main feature of YNS diagnosis. Frequently, all nails are affected with different degrees of severity. Manifestations typically include yellow to brown discoloration, attributed to the accumulation of lipofuscin pigment, along with impaired nail growth and onycholysis. The nail plate often appears thickened with increased transverse curvature and loss of the cuticle. Due to subungual hyperkeratosis, the nail becomes opaque, and the lunula is commonly absent. Additionally, erythema of the proximal nail fold may be observed, frequently in association with chronic paronychia [15]. In this case, both fingernails and toenails were affected, with all nails exhibiting dystrophy, varying degrees of discoloration, and absence of the cuticle.

Respiratory tract involvement occurs in more than 50% of patients and may present in different forms. The most frequently reported symptom is chronic cough, occurring in 56% of the cases, as well as pleural effusion, which is observed in 14-46% of the patients [4,8]. The pleural fluid may exhibit different characteristics, most commonly being bilateral in 68.3% of the cases and showing exudative features in 95% [16]. In the present case, the patient had recurrent pneumonia, a clinical finding reported in up to 22% of patients in the literature. The infectious agents most commonly implicated in recurrent respiratory infections are the same as those found in community-acquired pneumonia [17].

Pulmonary function tests are usually normal or may reveal a restrictive pattern, often attributable to pleural effusions. However, in this case, the patient exhibited a mild obstructive pattern, a finding also reported by Piraccini et al. [4] in their cohort. Thoracic CT is the preferred imaging modality for evaluating the various radiologic manifestations of YNS.

Lymphedema is one of the cardinal signs included in the diagnostic triad of YNS, occurring in 29-80% of the reported series and potentially representing the initial manifestation in up to one-third of the cases [1]. It predominantly affects the lower limbs, typically in a bilateral distribution and confined to areas below the knees (Figure 2). Lymphedema results not only from lymphatic fluid accumulation but also from fibrosis and adipose tissue hypertrophy, due to fibroblast and adipocyte stimulation [18]. The most significant complication is cellulitis, while discomfort and cosmetic concerns also impact quality of life. The response to diuretic therapy is frequently poor [10].

Rhinosinusitis may be present in over 83% of cases, with the maxillary sinuses being the most frequently affected. Nasal symptoms may precede, coincide with, or appear years after the onset of nail changes. The clinical presentation is typically characterized by daily mucopurulent rhinorrhea, nasal obstruction, and frequent postnasal drip, possibly associated with headaches or recurrent facial pain [19].

A wide range of comorbidities has been reported in association with YNS, such as acquired immune deficiency syndrome, malignancies, autoimmune, inflammatory, and hematologic diseases and immune deficiencies. The spectrum of associated diseases is broad and appears to lack a unifying pathophysiological mechanism, leading to the assumption that the occurrence of YNS in these patients may be coincidental [10].

The differential diagnosis of nail discoloration should include a history of pharmacological agents, such as D-penicillamine, bucillamine, and tiopronin; infectious diseases, including fungal infections, as well as chronic paronychia in the case of fingernail involvement and onychomycosis in toenails [1,4].

There is no established specific treatment for YNS; current therapeutic approaches are primarily symptomatic, except in cases where an identifiable underlying etiology allows for targeted management [20]. Patients with idiopathic YNS may benefit from a trial of medical therapy, typically initiated with oral vitamin E (1,000-1,200 IU/day), octreotide, and the use of compression stockings. In cases unresponsive to vitamin E monotherapy, adjunctive treatment with oral antibiotics, antifungals, or zinc may be considered. Invasive interventions, such as chemical pleurodesis, are reserved for refractory pleural effusions [1,10].

Although the prognosis of YNS is generally favorable, with spontaneous remission in up to 30% of patients [1], larger-scale studies are needed to strengthen the evidence and define the best diagnostic and therapeutic strategies, due to the increased number of cases reported in the literature.

## Conclusions

YNS is a rare and heterogeneous clinical entity, characterized by the triad of nail dystrophy, lymphedema, and respiratory involvement, though these features often develop asynchronously. The present case illustrates the diagnostic challenges of YNS, given its nonspecific and overlapping manifestations with autoimmune, infectious, and pulmonary diseases. Early recognition is essential to optimize supportive care, prevent complications, and guide multidisciplinary monitoring, particularly in patients with potential autoimmune serological findings. Although therapeutic options remain largely symptomatic, some patients may experience improvement with vitamin E supplementation and macrolide prophylaxis, as observed in this case. Given the limited understanding of its pathogenesis and the absence of standardized treatment protocols, further research and longitudinal studies are warranted to elucidate disease mechanisms, establish clear diagnostic criteria, and define evidence-based management strategies.
